# Case report: When infection lurks behind malignancy: a unique case of primary bone lymphoma mimicking infectious process in the spine

**DOI:** 10.3389/fnume.2024.1402552

**Published:** 2024-07-15

**Authors:** Ayoub Jaafari, Ornella Rizzo, Sohaïb Mansour, Anas Chbabou, Anne-Laure Trepant, Rachid Attou, Celine Mathey

**Affiliations:** ^1^ Department of Nuclear Medicine, H.U.B Erasme Hospital, Brussels, Belgium; ^2^ Department of Haematology, H.U.B Bordet Hospital, Brussels, Belgium; ^3^ Department of Internal Medicine, C.H.U Brugmann, Brussels, Belgium; ^4^ Department of Radiology, C.H.U Saint-Pierre, Brussels, Belgium; ^5^ Department of Anatomopathological, H.U.B Erasme Hospital, Brussel, Belgium; ^6^ Department of Intensive Care Unit, C.H.U Brugmann, Brussels, Belgium

**Keywords:** primary bone lymphoma, diffuse large B-cell lymphoma, spondylodiscitis, ^18^FDG-PET/CT, MRI

## Abstract

Primary bone lymphoma of the spine (PBL) is a rare entity that may be misdiagnosed due to its atypical location and clinical and imaging features mimicking certain pathologies as infectious processes, which complicates and delays diagnosis. Our case reports a patient in her sixties who had been suffering from chronic low back pain for a year, and had gradually started to develop cruralgia. She underwent a blood sample, magnetic resonance imaging (MRI), and positron emission tomography (^18^F-FDG-PET/CT) which revealed inflammatory syndrome, and an image of spondylodiscitis of the lumbar spine associated with a morphological and metabolical widespread invasion posteriorly suggesting epiduritis. No other lesions were found on the rest of the body. Neurosurgical management was performed and a biopsy was made. Histological results showed aggressive and diffuse large B-cell lymphoma, suggesting a diagnosis of PBL. This case highlights the first case of spondylodiscitis mimicking PBL in the lumbar spine, the intricacies of the diagnostic work-up, and the complexity of discriminating with an infectious process in the spine, as both have a similar, non-specific clinical presentation, while morphological and metabolic findings can be alike.

## 1 Introduction

First described by Oberling et al. in 1928, primary bone lymphoma in the spine (PBL) remains a scarce entity and the utmost shared type of non-Hodgkin lymphoma (LNH) appearing in bone lesions deprived of nodal or extra-nodal involvement, accounting for approximately 3%–7% amongst primary bone tumours and 2% of all lymphomas (1). The predominant diagnosis age of patients is over 30, with a median of 45, and more predominant in men than women, with a ratio of 1.5:1 (1). Most PBL are diffuse large B-cell lymphomas (DLBCL) and they commonly involve appendicular skeleton (2). Because infection and malignant tumours have certain characteristics in common that complicate and delay diagnosis, PBL can be underdiagnosed, particularly with tumours invading the spine/spinal cord or nerves such as sarcoma, plasmacytoma, or infectious processes such as osteomyelitis or spondylodiscitis. Here, we present the first unique case of an atypical cruralgia in terms of tumour location and clinical presentation, the ^18^F-FDG-PET/CT and MRI findings, and therapeutic management.

## 2 Case presentation

**Figure 1:**
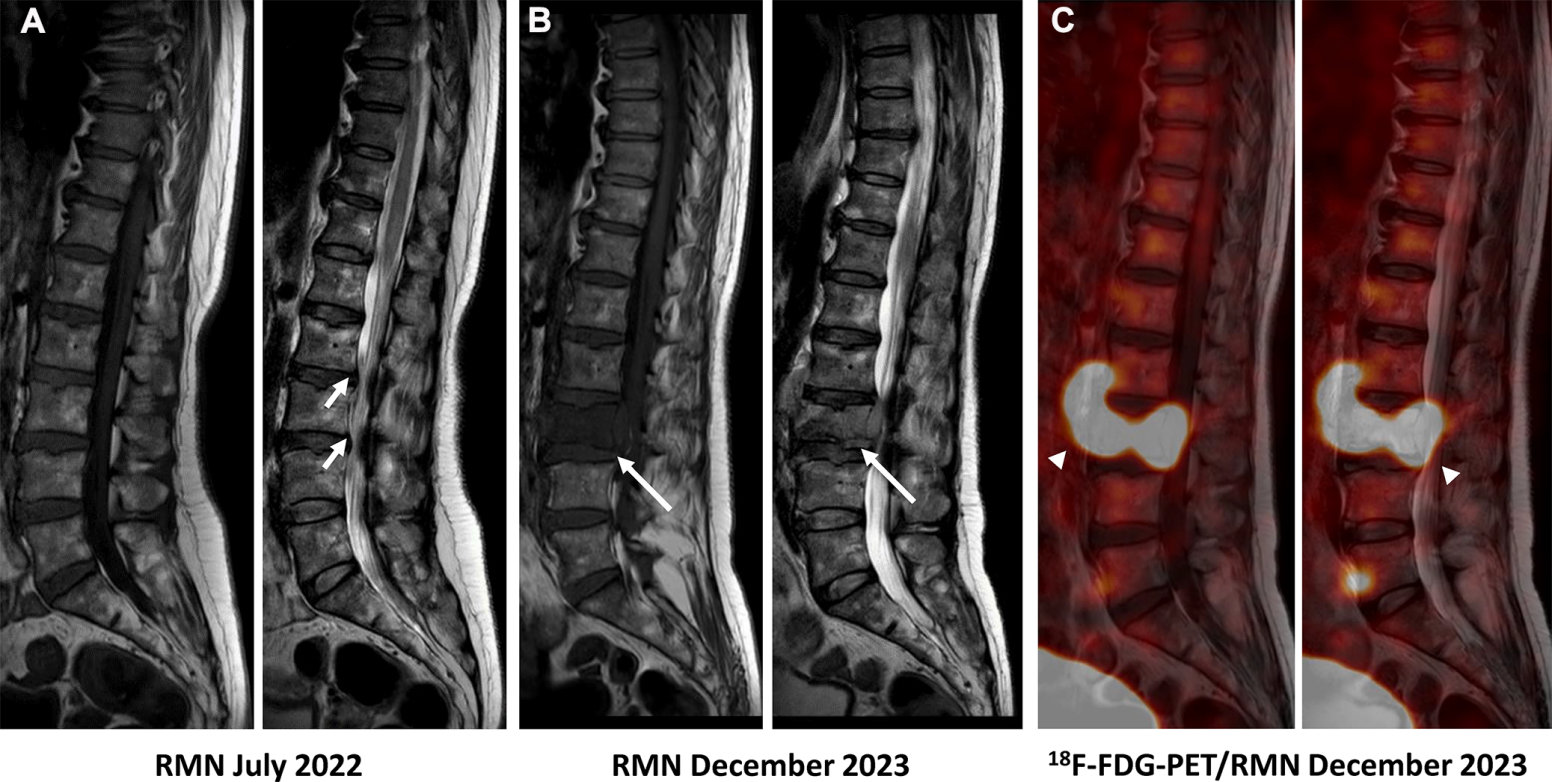
MRI (T1–T2) undergone in July 2022 showed disc disease in L2–L3 and especially L3–L4 (wihte arrow), the latter discreetly reducing the diameter of the canal, with a relative secondary narrowing of the canal. (**A**) The MRI carried out in December 2023 showed bone marrow replacement almost the entire L3-vertebral body in both phases (long white arrow), and an inflammatory/infectious spindle extending anteriorly and posteriorly, with suspected epiduritis. (**B**) A merged image of ^18^F-FDG-PET and MRI showed aggressive widespread invasion at the L3 and doubtful at the L3–L4 level intervertebral disc, with an onfectious appearance and metabolic extension anteriorly and posteriorly (head arrow), associated with epiduritis (**C**).

**Figure 2:**
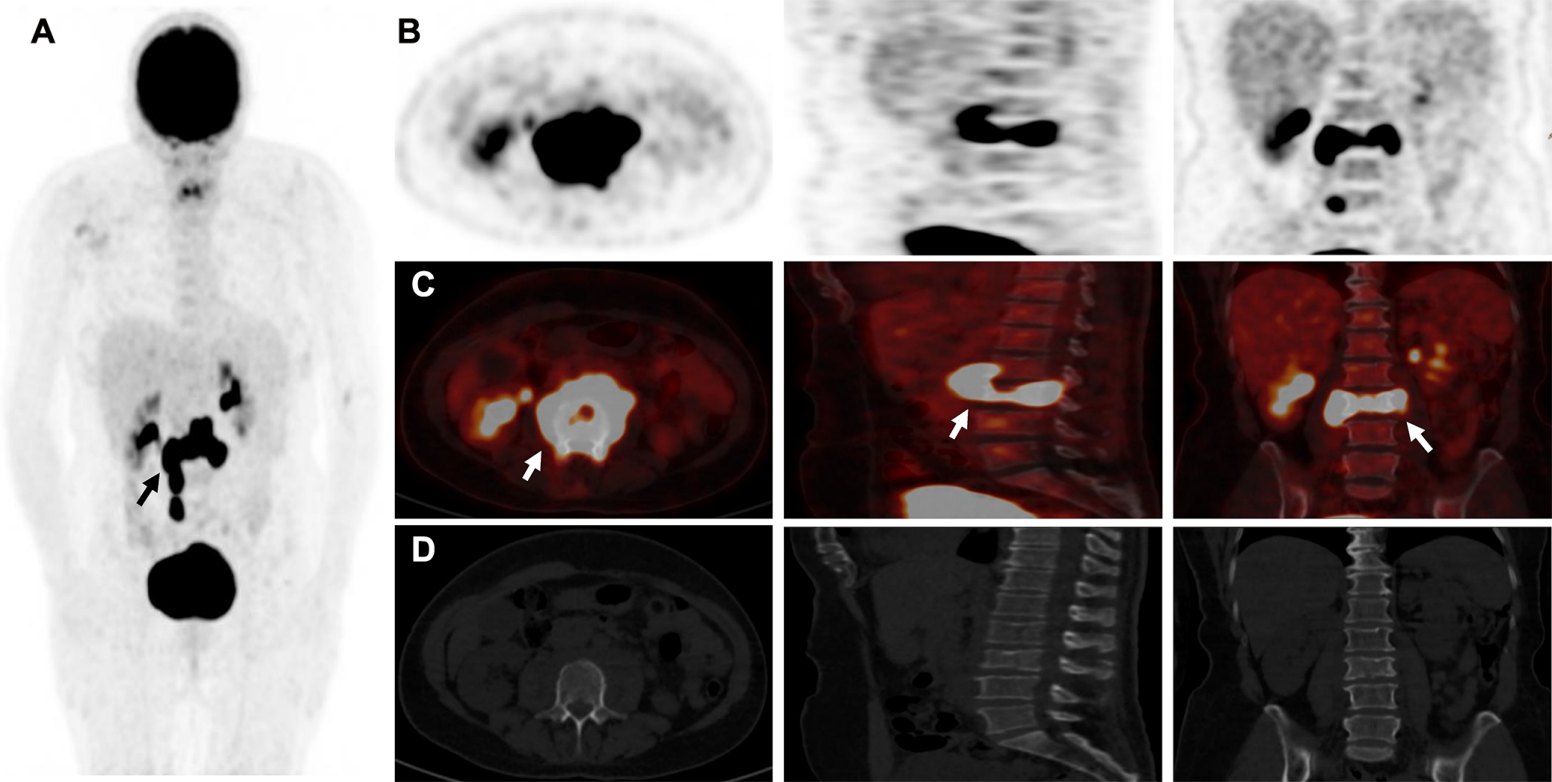
^18^F-FDG-PETCT showing higher uptake and extensive invasion at the L3 vertebral level and questionable invasion at the L3-L4 intervertebral disc level (white arrow), with an “inflammatory cast” appearance (black arrow) and more aggressive metabolic extension anteriorly and posteriorly in maximum intensity projection (MIP) (**A**,**B**) and merged images (**C**) than CT (**D**) in addition, no other lesions were found in the rest of the body explored.

**Figure 3:**
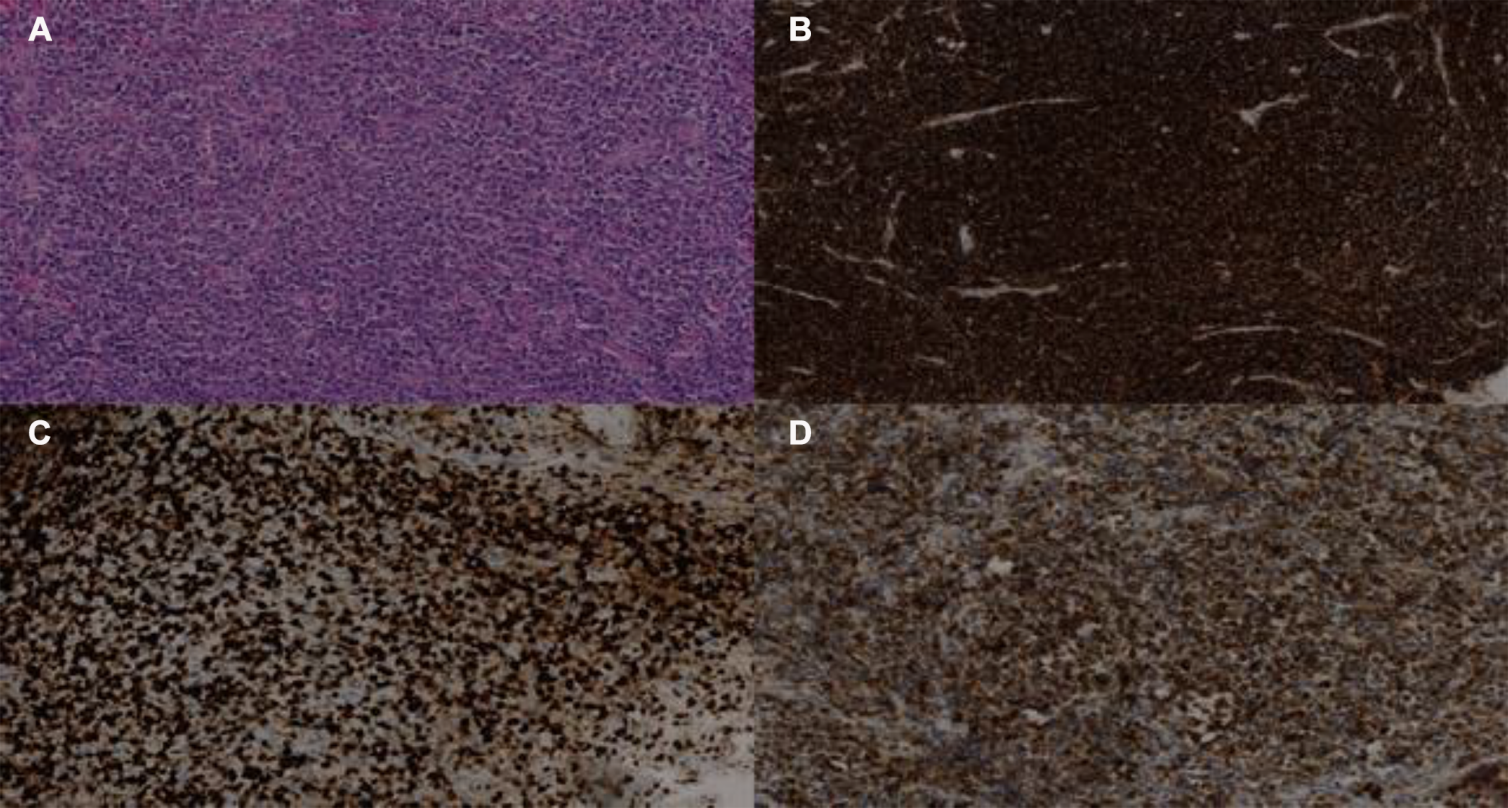
Biopsy of infiltrating lesion of the L3 vertebra showing aggressive DLBCL. Immunostaining of the lesion is positive for haematoxylin-eosin HE (**A**), CD20+ (**B**), KI-67 (**C**), and BCL2 (**D**).

**Figure 4:**
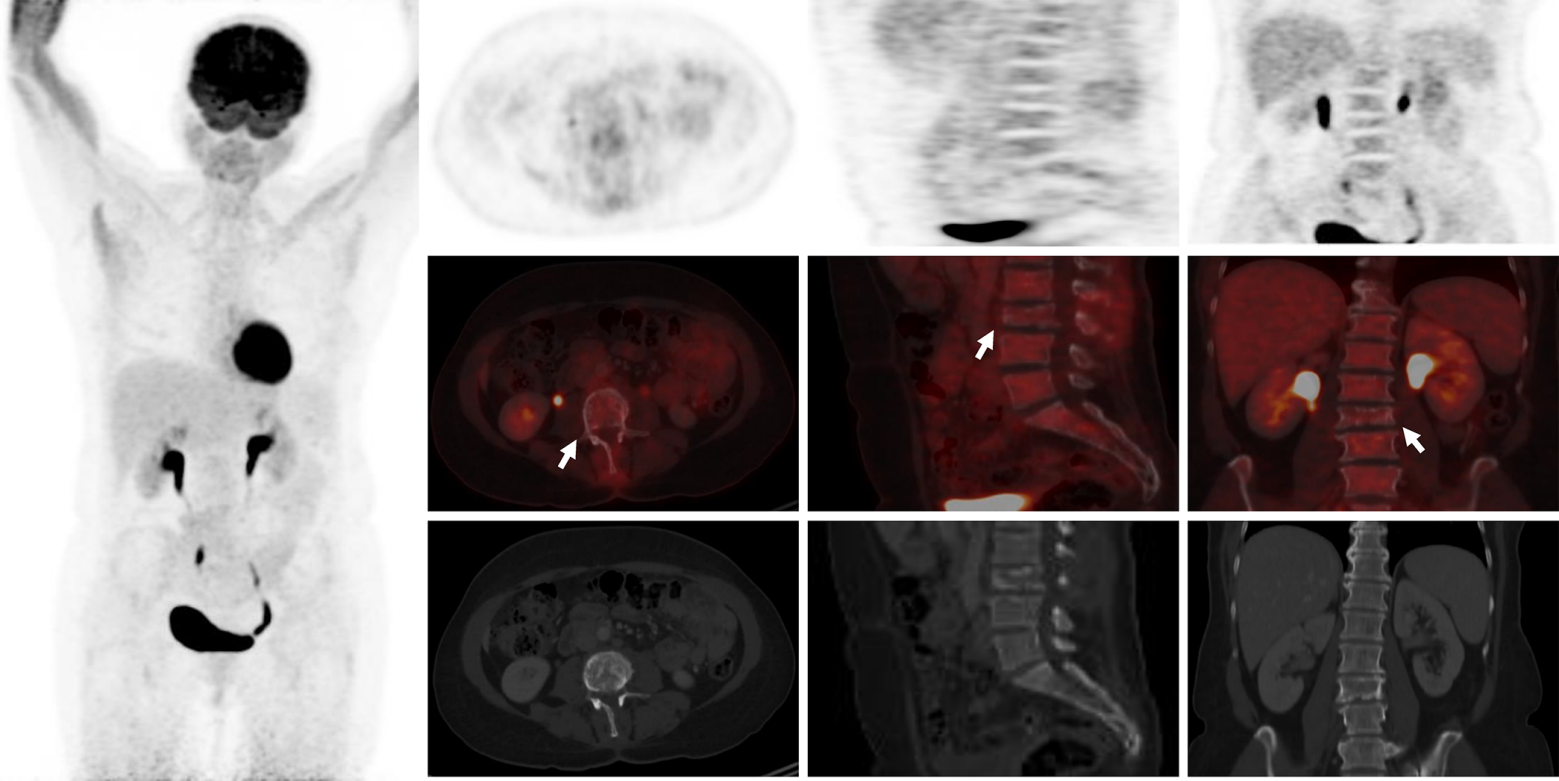
^18^F-FDG-PET/CT showing complete metabolic response/normalization at L3 vertebra level and the L3–L4 intervertebral disc after 4 cycles of chemotherapy (white arrows).

**Figure 5:**
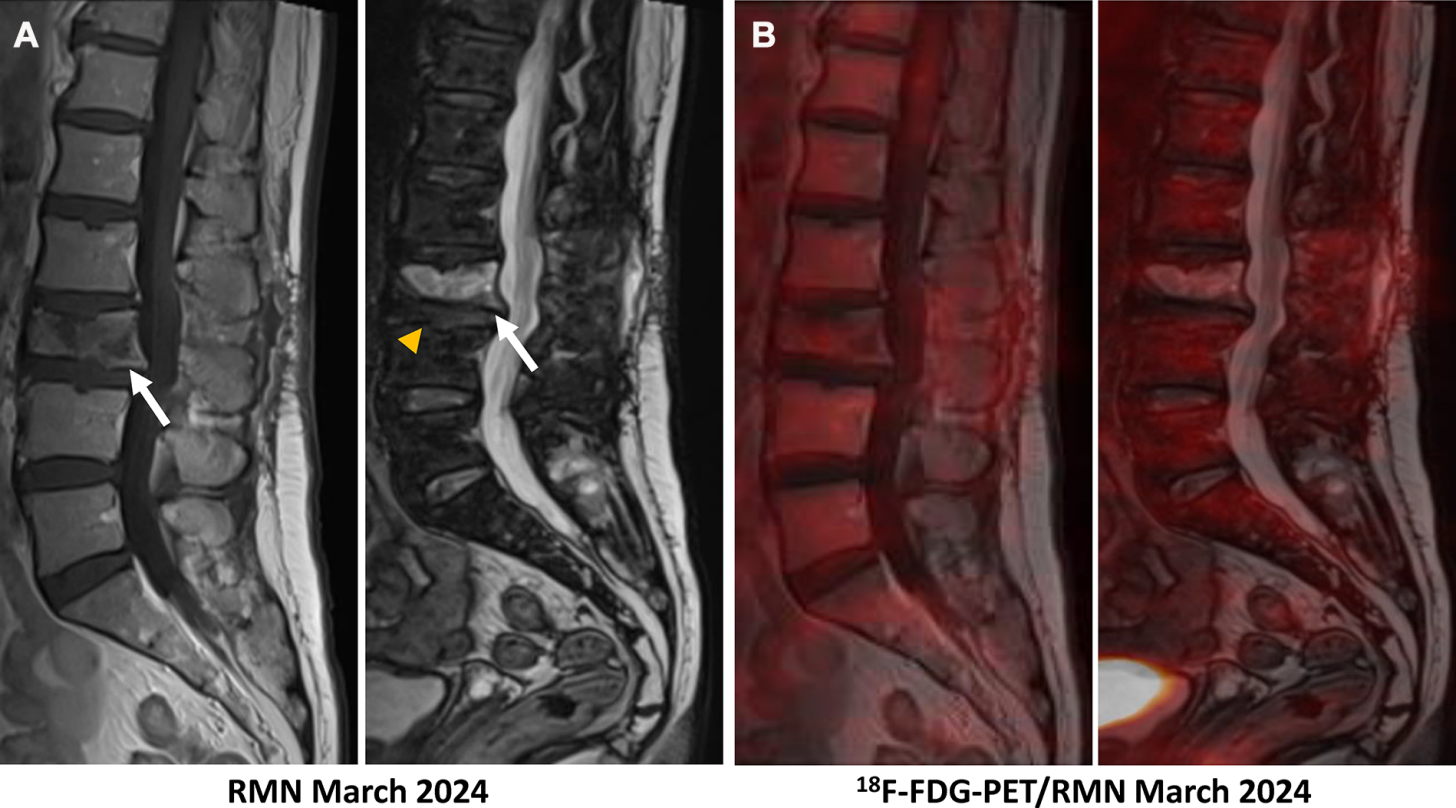
(**A**) The follow-up MRI realized in March 2024 showed traces of the laminectomy performed on L3, a clear reduction in tumour infiltration of the vertebral body of L3 in both phases (hypo-signal in T1 and hypersignal in T2–phase, white arrows) with pathological compaction (lost of height: 10 mm), and a reduction in the inflammatory spindle which extended anteriorly and posteriorly. The L3–L4 intervertebral disc remains reshaped but no longer shows associated enhancement (yellow head arrow). (**B**) A merged image of ^18^F-FDG-PET and MRI showed a complete metabolic response at the L3 vertebra and L3–L4 intervertebral disc after 4 cycles of chemotherapy.

A 62-year-old patient without a significant pre-existing medical history presented with chronic mechanical low back pain since the summer of 2022. She had previously undergone an MRI of the spine in favor of a narrow lumbar canal (Figure 1A), for which she was treated with multiple analgesics and infiltrations until the summer of 2023, with an improvement of the symptomatology. However, since October 2023, her symptoms have worsened. The patient presented no nocturnal sweating, was apyretic, and slightly decreased her appetite. On clinical examination, discrete paresis of the left quadriceps was observed. Bearing in mind the patient's condition at the time of consultation, she was quickly admitted to the hospital where further exams were carried out. Laboratory tests showed a small elevated serum white-blood cells (12.2 × 10^3^ /µl), predominantly neutrophilic (8.4 × 10^3^ /µl), and a concentration of C-reactive protein (75 mg/dl). The other biological tests came back unremarkable. She underwent an MRI, which revealed a very dubious image showing remodelling of the L3 vertebra (bone marrow replacement) and of the intervertebral disc between L3 and L4, suggesting suspected spondylitis vs. spondylodiscitis (Figure 1B). In view of her symptoms and recent weight loss, she also underwent an ^18^F-FDG-PET/CT showing a higher uptake in this widespread invasion at the L3 level with an infectious appearance and more aggressive metabolic extension anteriorly and posteriorly, associated with epiduritis (Figure 2). No other lesions were found on the rest of the body. Neurosurgical management (decompression and L3-laminectomy) was performed and samples were taken. Histological results showed aggressive and diffuse large-cell B lymphoma (DLBCL), stage IV (Ann-Harbor classification). Immuno-histochemical were as follows: CD20+, PAX5+, CD3/CD5 -, BCL6+, MUM1-, Cycline D1-, BCL2+, cMyc-, Ki-67 80%, consistent with DLBCL, non-germinal center B-cell like (Figure 3). A diagnosis of primary bone lymphoma of the lumbar spine was made. Chemotherapy (R-CHOP and methotrexate) was administered to the patient with clinical, morphologic, and metabolic improvement after 4 cycles (Figures 4, 5).

## 3 Discussion

The non-specific clinical manifestations and inconclusive laboratory tests of primary bone lymphoma (PBL) can be quite frustrating, and heterogeneous in its presentation, frequently resulting in a delayed diagnosis (2). Unlike lymph node lymphomas, which are confined to the lymphatic system, extra-nodal lymphomas can originate in various tissues and organs such as the gastrointestinal tract, skin, central nervous system, lungs and bones (2). The most common symptom is localized bone pain, predominantly in the appendicular skeleton, which is difficult to relieve with rest and may progressively worsen over time (3). Patients may sometimes experience “B” symptoms including fever, night sweats, and unwanted weight loss. These symptoms occur in a minority of patients and are not as frequent in PBL as in systemic lymphoma (4). In our case, the patient reported a “red flag” sign (unwanted weight loss) associated with cruralgia and inflammatory syndrome, suggesting an infectious process on the first hypothesis. PBL can arise in any part of the musculoskeletal system and can occasionally manifest as soft tissue masses that can be misidentified for infection or malignancy (5, 6). Moreover, symptoms of spinal cord compression are reported in approximately less than 20% (5, 6).

According to the literature, this paper reported the first case of PBL mimicking an isolated infectious process in the spine. As far, few case reports of PBL infiltrated spinal nerve roots and ganglia have been described but nothing related to our case (7, 8).

Since infections and malignant tumours share characteristics that complicate the differential diagnosis and delay appropriate treatment, multiple modalities including clinical examination, laboratory sampling, biopsy samples, and imaging techniques are required.

In morphological imaging, magnetic resonance imaging (MRI) has been proven to be significant in the staging of PBL (9). However, MRI cannot discriminate non-viable from viable malignancies as shown in our patient (Figure 1B), and therefore cannot be convenient where bone infections are present. In contrast, MRI is currently the method of choice for diagnosing spondylodiscitis, demonstrating a hyposignal in T1 and a hypersignal in T2 phase (Figure 1B) (10). It provides information on bone marrow, vertebral disks, and neural structures.

In metabolical imaging, management with 2-[18F]-fluoro-2-deoxy-D-glucose (^18^F-FDG) positron emission tomography combined with computed tomography (PET/CT) is mainly used to identify high-uptake lesions. This hybrid imaging method provides information on metabolic characteristics and anatomical location. It has been approved for the diagnostic, staging, and therapeutic follow-up of patients with Hodgkin's disease (HD) and NHL (11). Study data with PBL also speculated that ^18^F-FDG-PET/CT may provide significant information for management decisions (11, 12). In spondylodiscitis, some studies have suggested that ^18^F-FDG-PET/CT is slightly more sensitive and accurate than MRI, particularly in the early stages, because the metabolism of the lesion appears much earlier than the morphological alterations on MRI (12, 13).

Nevertheless, discrimination between malignant and infectious processes is sometimes challenging with ^18^F-FDG-PET/CT. While maximum higher standardized uptake values (SUVmax) are associated with a greater probability of malignancy, no threshold for uptake allows us to distinguish both processes (14). For example, a prospective study conducted by Skawran et al. evaluated 77 cancerous and inflammatory/infectious lesions in 24 patients using different ^18^F-FDG-PET/CT parameters and showed no significant difference between infectious from malignant lesions using SUVmax (15).

In our case, the patient had no risk factors for spondylodiscitis (no recent spinal surgery, diabetes, pre-existing infection, iatrogenic or human immunosuppression, etc.), and the weight loss and cruralgia didn't appear until very late (1 year). It is possible that the various lumbar infiltrations carried out to relieve the pain served as an entry point for a bacterium that could have proliferated, giving rise to the initial suspicion of spondylodiscitis. The ^18^F-FDG-uptake in the L3-lesion was very intense (SUVmax 35) and correlated with a metabolic image of anterior and posterior “inflammatory cast” invading the spinal cord, suggesting an infectious origin as a first hypothesis but which was misdiagnosed in favor of an aggressive LDBCL. Clearly, in most cases, biopsy or pathology results are required to confirm the final diagnosis.

More recently, interest in the ^18^F-FDG-PET/MRI hybrid imaging has grown. This new modality combines the physiological information acquired by PET with the very high resolution of soft tissue and the contrast of MRI, reducing the number of examinations required for the patient and scanner-related irradiation (Figure 1C) (13). In PBL, ^18^F-FDG-PET/MRI has shown encouraging results in early studies and exposed similar sensitivity with ^18^F-FDG-PET/CT (16). In vertebral osteomyelitis, a prospective study conducted by Kouijzer et al. showed promising results as a complementary fusion-imaging technique with ^18^F-FDG-PET/MRI (17). Consequently, more studies are needed to fully demonstrate its benefit.

Treatment strategies depend on the type, location and stage of the lymphoma. Common treatments include chemotherapy, radiotherapy, immunotherapy and, in some cases, surgery (18). For some types, targeted therapies and haematopoietic stem cell transplants may be considered. The prognosis of extra-nodal lymphomas varies according to the subtype of lymphoma and its location (18). Because PBLs are often DLBCLs, the combination of Rituximab, cyclophosphamide, doxorubicin, vincristine, and prednisone (R-CHOP) associated with methotrexate for preventing central nervous system (CNS) is the most current protocol therapy for patients with aggressive DLBCL (19). Among patients who respond successfully to treatment, ^18^F-FDG-PET/CT shows a rapid decrease in FDG-uptake compared with baseline imaging, and any new FDG-avid lesion identified is considered to be a recurrence (20). ^18^F-FDG-PET/CT can therefore contribute to the management of patients with primary bone lymphoma. Our patient underwent 4 cycles of chemotherapy with an improvement in her symptomatology and morphological and metabolical imaging (Figures 4, 5).

## 4 Conclusion

Our case illustrates the difficulty of discriminating between primary bone lymphoma in the spine and spondylodiscitis, as both have a similar, non-specific clinical and laboratory presentation, while morphological and metabolic findings may be similar to each other. Diagnosis typically involves imaging studies, biopsies, and histopathological examination to confirm the presence of lymphoma cells. Treatment strategies depend on the type, location, and stage of the lymphoma. Common treatments include chemotherapy, radiotherapy, immunotherapy, and, in some cases, surgical intervention. For certain types, targeted therapies and hematopoietic stem cell transplantation may be considered. For precise diagnosis, we recommend fully incorporating PBL as a differential diagnosis in patients presenting with an inflammatory syndrome associated with neurological defects and warning signs, and combining clinical diagnosis, laboratory tests, fusion imaging methods, and biopsies to avoid delaying appropriate treatment.

## Data Availability

The datasets presented in this study can be found in online repositories. The names of the repository/repositories and accession number(s) can be found in the article/Supplementary Material.
